# Editorial: Measurement in Health Psychology

**DOI:** 10.3389/fpsyg.2022.937700

**Published:** 2022-05-24

**Authors:** Giulia Casu, Antonio de Padua Serafim, Victor Zaia, Paola Gremigni

**Affiliations:** ^1^Department of Psychology, University of Bologna, Bologna, Italy; ^2^Institute of Psychology, University of São Paulo, São Paulo, Brazil; ^3^Postgraduate Program in Health Sciences, Centro Universitário FMABC, Santo André, Brazil

**Keywords:** Health Psychology, measurement, health outcomes, validation, psychometrics

According to the American Psychological Association (APA, 2003; Freedland, 2021), Health Psychology is an interdisciplinary subspecialty of Psychology concerned with the study of biological, behavioral, and social factors contributing to both health and illness. Health Psychology applies its principles, techniques, and scientific knowledge to evaluate, diagnose, treat, modify and prevent physical, mental, or any other problems relevant to the processes of health and disease. It focuses on the promotion and maintenance of health, the prevention and treatment of illness, and the identification of etiologic and diagnostic correlates of health, disease, and dysfunctions.

Several issues and factors are still understudied in Health Psychology. Psychological effects of the COVID-19 outbreak, caregiving experiences, work-related stress and life-work balance, quality of life of sexual minorities, health-related orientations and motivations among younger generations, and the impact of the new media on people's mental health are just a few examples of Health Psychology phenomena that need further investigation. A critical issue in Health Psychology is measurement. A variety of crucial psychological constructs, health-related behaviors, and responses to health, illness, and healthcare need to be addressed and measured in Health Psychology research and practice. Intervention programs designed to foster good health by changing negative health behavior, promoting positive health behavior, and enhancing the management of chronic conditions have been of particular interest to Health Psychology since its beginnings as a distinctive discipline. The availability of psychometrically sound measures is crucial to assessing the effectiveness of such interventions and deepening our understanding of the social and psychological processes of health and illness (Apple, 2005; Etches et al., 2006).

We launched the Research Topic “Measurement in Health Psychology” in this context. Because measures are a critical part of research and practice in Health Psychology, we were interested in providing an overview of up-to-date measurement principles and methods, which could help improve the process of developing valid, reliable, and sensitive instruments to be used in the fields of Health Psychology.

Within this Research Topic, we brought together research studies developing new tools or advancing the psychometric study of existing measures relevant to Health Psychology. Twenty-three articles authored by 105 contributors from different countries and continents were accepted. The research contributions published in this Research Topic relate to self-report measures of socio-environmental and psychological/behavioral influences on health and disease, as framed within the biopsychosocial model (Engel, 2012), which has traditionally guided the field of Health Psychology, and the study of health outcomes.

## Socio-Environmental Influences on Health and Disease

Six articles in this Research Topic addressed constructs belonging to the social domain of the biopsychosocial model. Three papers dealt with the assessment of family relationships. One of them (Zhan and Wang) presented the development of a new measure to assess the subjective evaluation of harmonious family relations as a resource for physical and mental health. Three independent samples of Chinese university students were enrolled, respectively, for item analysis and exploratory factor analysis (EFA) of the initial item pool; confirmatory factor analysis (CFA) and assessment of internal consistency; and relationships to other variables and test-retest reliability over a two-month interval. Results supported a nine-factor model of family harmony, with adequate reliability and validity as evidenced by positive correlations with criterion measures of subjective well-being and family function, cohesion, and adaptability, and negative correlations with loneliness scores.

Another paper (Guo et al.) evaluated the psychometric properties of a Chinese version of the 10-item Family Communication Scale (FCS) to measure positive family communication. EFA and CFA supported a one-factor structure, which showed adequate internal consistency and test-retest reliability and correlated in the expected direction with wellbeing indicators and frequency of communications with family members via Information and Communication Technologies.

Finally, UK researchers (Bywater et al.) proposed a new short measure to assess the bonding between parents and children under 1 year of age, namely the “Me and My Baby” (MaMB) questionnaire. Factor analyses and Rasch calibration performed on data from 434 mothers provided initial evidence that the MaMB reliably measures infant bonding.

In the context of caregiving, one paper tested the psychometric characteristics of the Zarit Burden Interview in Peruvian informal primary caregivers of persons diagnosed with intellectual disabilities (Boluarte-Carbajal et al.). Applying CFA and Rasch analysis, the authors found evidence of a unidimensional structure with adequate reliability. Evidence of validity was provided by relationships with a measure of the risk of physical and psychosocial abuse and neglect by primary caregivers. As for formal caregiving, Italian researchers developed a 20-item scale to assess emotional, informational, appraisal, and instrumental social support by healthcare providers in the oncology setting (Tomai and Lauriola). Scale dimensionality and reliability were tested using exploratory structural equation modeling (ESEM) and Mokken scaling analysis. Evidence of validity and reliability and expected associations with doctor communication skills and trust in the physician supported the use of this new measure of healthcare social support as multidimensional a construct.

Finally, as for the school environment, one article (Carmona-Halty et al.) tested the validity and reliability of the School Burnout Inventory among Chilean high school students. CFAs on the 8-item Chilean version supported two statistically equivalent first- and second-order three-factor models of school burnout as composed of exhaustion, cynicism, and inadequacy, which showed gender invariance. Internal consistency and test-retest reliability were acceptable, and school burnout scores correlated as expected with study-related emotions, academic psychological capital, and academic engagement.

## Psychological/Behavioral Influences on Health and Disease

Nine papers addressed the assessment of individual characteristics and behaviors relevant to health and illness. Within the field of psychosomatic disorders, Iranian researchers (Lashkari et al.) tested the psychometric properties of the Farsi version of Perth Alexithymia Questionnaire (PAQ). Based on CFA results, the 5-factor model of the original PAQ replicated well on data from college students, with adequate internal consistency and test-retest reliability. Evidence of associations with other variables (TAS-20, emotion regulation, depression, anxiety) was collected for the Farsi PAQ.

The Work-Related Rumination Scale was tested on a sample of Puerto Rican workers (Rosario-Hernandez et al.). An 11-item Spanish version with three factors (affective rumination, problem-solving pondering, and detachment) was obtained using CFA and ESEM and proved to be invariant across gender and age within and between five different study samples. Reliability coefficients were satisfactory, and correlations with relevant criterion measures (e.g., sleep quality, emotional exhaustion) provided evidence of convergent and divergent validity.

As to individual beliefs and expectations, one article (Lang and Ye) presented a Chinese adaptation of the Self-Objectification Beliefs and Behaviors Scale (C-SOBBS). As a result of exposure to sexual objectification in interpersonal situations and visual media, self-objectification entails viewing one's own body from a third person's perspective and has been linked to poorer women's mental health. The authors found evidence of structural (CFA), convergent, discriminant, and incremental validity, and adequate internal consistency and test-retest reliability for the C-SOBBS. In another paper, Chinese researchers proposed the development and validation of the Psychological Needs of Cancer Patients Scale to identify the psychological care demands of cancer patients (Chen et al.). Results of EFA and CFA supported a six-factor model of value and esteem, independence and control, mental care, disease care, belonging and companionship, and security, with acceptable reliability and expected associations with anxiety and depression.

Two articles addressed situation-specific coping. The Robust Pandemic Coping Scale (R-PCS) was developed to assess coping strategies related to pandemic situations at all stages of the epidemic management cycle (Burro et al.). Data from Italian university students were analyzed via EFA and CFA, followed by Rasch analysis. A four-factor model of despair, adjustment, proactivity, and aversion was supported, which was invariant across gender and age and showed adequate reliability. Discriminant and criterion-related validity based on correlations with personality characteristics helpful in coping with disasters and predictive validity on levels of enjoyment and anger 2 months later were also supported. To assess coping strategies to deal with cancer, researchers from Portugal (Lemos et al.) performed a cross-cultural adaptation and psychometric evaluation of the Perceived Ability to Cope with Trauma Scale (PACT). Results of CFA on data from patients recently diagnosed with early breast cancer supported the original PACT two-factor model of coping flexibility as composed of forward and trauma focus domains, which showed adequate internal consistency and associations in the expected direction with self-efficacy to cope with cancer, quality of life and psychological distress.

In the area of health-related attitudes, Chilean researchers (Ferrer-Urbina et al.) developed a brief scale to assess the affective, cognitive, and behavioral attitudes of youth and young adults toward condom use. Using EFA and ESEM, the authors found support for the hypothesized three-factor model. The scale showed strong invariance across gender, adequate reliability, and expected relationships with sexual risk behaviors and condom use.

Among health-related behaviors, one study (Milasauskiene et al.) tested the psychometric performance of a very brief measure of problematic internet use, namely the Compulsive Internet Use Scale (CIUS), when used with Lithuanian medical students and resident doctors. Results indicated that the brief, 5-, 7-, and 9-item versions of the CIUS were reliable and valid screening tools to assess the severity of symptoms of problematic internet use in the medical population. Another brief, 6-item scale of excessive use of social networks was adapted for use with Mexican adolescents and young adults (Salas-Blas et al.). Altogether, the structural properties of the response options fitted the partial-credit model. CFA supported a single domain of addiction to social networks, with measurement invariance across sex, age, and educational campus, good internal consistency, and theoretically consistent associations with sensation seeking and depression.

## Health Outcomes

A total of eight papers focused on assessing outcomes of the interactive relationships between biological, environmental, and psychological/behavioral factors. Two papers focused on health-related quality of life (HRQoL), which is a primary outcome in the evaluation of interventions' effectiveness on people's health. One article (Xu et al.) presented the traditional Chinese version of the Recovering Quality of Life (ReQoL) outcome measure. The ReQoL measures mental health recovery, defined as a self-directed process of healing and transformation, which has received increasing attention in evaluating the outcomes of mental care. The Chinese ReQoL showed good psychometric properties in terms of internal consistency, test-retest reliability, factor structure (CFA), known-group validity, and associations with relevant variables in the general population.

Lithuanian researchers (Gecaite-Stonciene et al.) examined the validity and reliability of the Minnesota Living with Heart Failure Questionnaire in individuals with coronary artery disease. EFA and CFAs supported a three-factor model of physical, social, and emotional disease-specific HRQoL. The Lithuanian version was reliable and showed evidence of convergent validity based on correlations with another measure of HRQoL (SF-36) and with exercise capacity assessed using a standardized computer-driven bicycle ergometer.

Six papers addressed the assessment of indicators of mental health or adjustment to health problems. One of them presented the validation of a Kazakhstani version of the Mental Health Continuum–Short Form to assess emotional, social, and psychological wellbeing (Hernandez-Torrano et al.). CFA confirmed a bifactor model, which was invariant across gender and age in a sample of university students. Based on reliability analyses, the authors advised against the interpretation of specific-factor scores and recommended the computation of a general wellbeing score.

Another article (Flenreiss-Frankl et al.) presented the validation study of a multidimensional inventory for the assessment of mental pain after traumatic experiences (FESSTE30) in the German speaking general population. CFA showed a satisfactory fit of a five-factors measuring somatization, depression, intrusive memories, dissociation, and anxiety. The scale showed evidence of reliability and strong correlations with measures of psychological distress, PTSD symptoms, and the extent of traumatic experiences.

The Swedish version of the Multidimensional Inventory for Religious/Spiritual Well-Being (MI-RSWB) was psychometrically tested to assess the spiritual wellbeing of a large sample of university students (Wenzl et al.). Based on the results of PCA and CFA, the authors proposed a revised model (MI-RSWB-R) with general religiosity and connectedness domains reflecting religious and spiritual wellbeing, respectively, and suggested using the remaining subscales (immanent and transcendent hope, forgiveness, sense of meaning, and connectedness) to gain insight into specific, separate facets of wellbeing. All MI-RSWB-R domains showed adequate reliability and relationships with the centrality of religiosity.

Brazilian and Italian researchers (Casu et al.) tested a Brazilian-Portuguese version of the 12-item Infertility-Related Stress Scale (IRSS-BP) to assess adjustment to the infertile condition. ESEM showed a bifactor model with one general and two specific intrapersonal and interpersonal stress factors, which was invariant across Brazilian and Italian infertile individuals. All three IRSS-BP factors showed adequate composite reliability and theoretically meaningful associations with gender, infertility duration, and depression scores.

Using Rasch's partial-credit model, correlation, and regression, one study (Hum et al.) tested the psychometric properties of the Starkstein Apathy Scale (SAS) to assess apathy in a sample of English speaking people having experienced a stroke. A revised 9-item version of the SAS targeting impairment of apathy/motivation was obtained, which was unidimensional and reasonably reliable, with no substantial item differential functioning across time, age, sex, and education.

Chinese researchers developed and tested a 40-item short form of the Inventory of Psychosocial Balance (CIPB-SF) to assess ego development based on Erikson's theory in Chinese older adults (Chen et al.). Ego integrity in older people is crucial to help them achieving successful aging and a better quality of life. Through a three-step process involving expert validity and piloting, item analysis and principal component analysis, CFA, and assessment of reliability and relations to criterion measures, the authors found acceptable validity and reliability evidence, supporting the use of the Chinese IPB.

## Conclusions

In this Research Topic, we invited researchers from around the world to contribute to measuring key constructs in Health Psychology by sharing the development and improvement of measures to be used by Health Psychology researchers and clinicians.

Two-thirds of the accepted papers presented further development and validation of existing instruments for use in different cultural contexts or populations with specific characteristics. This emphasizes that the validation process is never complete but is instead an ongoing process, which involves collecting multiple types of evidence to support the appropriateness and meaningfulness of inferences and decisions made from measurement scores (Messick, 1989). Most papers examined the factor structure of measures using Classical Test Theory (CTT). The remaining papers used both CTT and Item Response Theory models. Within CTT, EFA and/or CFA were the most common analytic choice. However, some contributors preferred to combine the advantages of both EFA and CFA and use ESEM. About half of the articles in this Research Topic presented the validation of very brief measures, which are particularly suitable for minimizing patient and staff burden and ensuring time-efficient assessments in busy health settings.

Altogether, 23 measures with promising psychometric characteristics are made available to researchers and clinicians to ensure a valid and reliable assessment of psychosocial variables and behaviors that may influence health and illness, as well as to evaluate health outcomes and monitor the effectiveness of Health Psychology interventions. This Research Topic certainly does not exhaust the issue of measurement in Health Psychology. Still, we hope that other colleagues will take new initiatives to further share the development and testing of valid, reliable, and sensitive instruments to be used in Health Psychology research and practice.

## Author Contributions

All authors listed have a substantial and equal contribution to the work and approved it for publication.

## Conflict of Interest

The authors declare that the research was conducted in the absence of any commercial or financial relationships that could be construed as a potential conflict of interest.

## Publisher's Note

All claims expressed in this article are solely those of the authors and do not necessarily represent those of their affiliated organizations, or those of the publisher, the editors and the reviewers. Any product that may be evaluated in this article, or claim that may be made by its manufacturer, is not guaranteed or endorsed by the publisher.
